# Coexistence of Peripheral Retinal Diseases with Macular Hole

**DOI:** 10.4274/tjo.galenos.2019.06706

**Published:** 2019-09-03

**Authors:** Erdoğan Yaşar, Nazmiye Erol, Mustafa Değer Bilgeç, Ayşe İdil Çakmak

**Affiliations:** 1 Aksaray University, Aksaray Training and Research Hospital, Department of Ophthalmology, Aksaray, Turkey; 2 Eskişehir Osmangazi University Faculty of Medicine, Department of Ophthalmology, Eskişehir, Turkey; 3 Mustafa Kemal University Faculty of Medicine, Department of Ophthalmology, Hatay, Turkey

**Keywords:** Macular hole, retinal tear, retinal hole, lattice degeneration

## Abstract

**Objectives:**

To investigate the frequency of retinal tear, retinal hole, and lattice degeneration in peripheral retinal examination of patients with macular hole.

**Materials and Methods:**

The files of patients who underwent pars plana vitrectomy surgery with a diagnosis of macular hole at Eskişehir Osmangazi University Department of Ophthalmology between 2008 and 2018 were retrospectively analyzed. A total of 106 patients with primary macular hole who underwent peripheral retinal examination were included in the study. The frequency of retinal tears, holes, and lattice degeneration associated with macular hole was investigated.

**Results:**

Peripheral retinal examination of 106 patients who underwent macular hole surgery revealed retinal tear in 3 patients (2.8%), retinal hole in 4 patients (3.8%), and lattice degeneration in 10 patients (9.4%). Retinal hole and lattice degeneration were observed concomitantly in 1 patient.

**Conclusion:**

This study showed that patients with macular hole have concomitant retinal tears and holes, which are also thought to arise due to vitreoretinal traction, at a frequency similar to that in the general population. This result suggests that both the anterior and posterior vitreous may have different pathologies at the same time related to these diseases.

## Introduction

Macular hole (MH) is a defect of the foveal neurosensory retina that is usually round in shape and includes all of the vertical retinal layers.^1^ Although numerous factors have been implicated in its etiology, perifoveal posterior vitreous detachment (PVD) is considered the most important, as it leads to vitreomacular traction (VMT) by exerting a dynamic force in the anteroposterior direction.^2,3^ Intraretinal pseudocysts develop following VMT, and these cysts merge and expand, transforming into a full-thickness hole.^3^ The prevalence of MH in individuals over 40 years of age is 0.1-0.8%, with women comprising two-thirds of patients.^4,5^ MH is associated with reduced visual acuity, metamorphopsia, micropsia, and rarely, photopsia.^6^ Optical coherence tomography (OCT) is the gold standard in diagnosis and follow-up.^7,^^8^ In surgery, tangential and anteroposterior vitreous traction on the macula is released and the detached retina adjacent to the hole is reattached. With posterior hyaloid and internal limiting membrane peeling and intravitreal gas tamponade, the success rate is 85-100%.^9,10^

Similar to MH, the prevalence of retinal tear associated with traction that occurs during PVD was found to be 1-3.3%, including the studies on postmortem eyes,^11,12,13,14^ while the prevalence of retinal hole was found to be 2.4-4.4%.^15,16^ In another study, the prevalence of retinal holes and tears was reported to be 2%, which was relatively less than in other studies.^17^ The prevalence of lattice degeneration was found to be 6-10.7%.^18,19,20^ Studies on the association of MH and peripheral retinal disorders are limited, but one study determined the total prevalence of retinal tear + retinal hole + lattice degeneration in patients with MH to be 33.8%.^21^

As these retinal defects are believed to be caused by vitreoretinal traction, similar to the etiopathogenesis of MH, the aim of our study was to determine the prevalence of retinal hole, retinal detachment, and peripheral retinal degenerations in MH patients in order to contribute to the literature regarding the need for peripheral retinal examination in this patient group.

## Materials and Methods

The study was conducted according to the principles of the Declaration of Helsinki, and ethics committee approval was obtained from Eskişehir Osmangazi University Ethics Committee. We retrospectively analyzed surgical notes from the files of patients who underwent pars plana vitrectomy surgery for a diagnosis of MH between 2008 and 2018 at Eskişehir Osmangazi University, Faculty of Medicine, Department of Ophthalmology. A total of 103 eyes of 103 patients who were diagnosed with primary MH and underwent peripheral retinal examination were included in the study. Those with a history of trauma, vitrectomy, panretinal laser therapy, degenerative myopia, any retinal disorder that may affect the peripheral retina, and those without a surgical note regarding the peripheral retinal screening during MH surgery were excluded. MH staging was based on fundus examination and optical coherence tomography results.^22^ According to the classification used, vitreopapillary traction was recorded as stage 1; MH ≤250 µm in diameter as stage 2; 250-400 µm MH as stage 3; and MH ≥400 µm as stage 4. In terms of PVD, those with stage 2 and 3 MH were considered incomplete PVD, and those with stage 4 MH were considered complete PVD. Retinal hole, retinal tear, and peripheral retinal degenerations detected via peripheral retinal examination with scleral indentation were recorded.

### Statistical Analysis

IBM SPSS for Windows version 22.0 software was used for statistical analyses. Using descriptive statistical analysis, numerical variables were presented as mean ± standard deviation. Binominal logistic regression analysis was used to assess correlations between variables. A p value <0.05 was considered statistically significant.

## Results

A total of 106 eyes of 106 patients were included in the study. The patients were between 41 and 87 years of age, with a mean age of 68.4±9.6 years. Sixty-six (62.2%) of the patients were female and 40 (37.8%) were male.

MH surgery was performed on the right eyes of 54 (50.9%) patients and on the left eyes of 52 (49.1%) patients. The surgery was done using 25-gauge vitrectomy in 49 (46.2%) patients, 23-gauge vitrectomy in 46 (43.4%) patients, and 20-gauge vitrectomy in 11 (10.4%) patients. We found that 10 of the 106 patients underwent simultaneous cataract and MH surgery.

Preoperative MH staging of the operated patients was recorded as stage 2 in 12 patients (11.3%), stage 3 in 77 patients (72.6%), and stage 4 in 17 patients (16.1%). In addition, 89 patients (84%) had incomplete PVD while 17 patients (16%) had complete PVD. 

The findings of peripheral retinal examinations performed at the end of MH surgery are summarized in Table 1.

According to these results, 16 (15.1%) of the 106 patients had peripheral retinal disorders, while 90 patients (84.9%) did not.

Binominal logistic regression analysis revealed no correlation between higher MH stage and retinal tear, retinal hole, or retinal degeneration (p>0.05). Of the 90 patients who had no accompanying peripheral retinal disorder, 55 (61.1%) were given SF6 gas and 35 (38.9%) were given C3F8 gas as intravitreal tamponade during MH surgery.

The other patients received intravitreal gas injection as well as 2-3 rows of endolaser around 9 lattice degenerations, 3 retinal holes, 1 lattice degeneration + retinal hole, and 3 retinal tears detected in the peripheral retina at the end of surgery.

## Discussion

Although numerous factors have been implicated in the pathophysiology of age-related primary idiopathic MH, VMT is considered the main etiology.^2,3^ Studies on MH have shown that its prevalence is higher among individuals 60-70 years of age and 2-3 times higher in women.^23,24^ In our study, the mean age of 106 patients who underwent MH surgery was 68.4±9.6 years, and the prevalence was higher in females (62.2%).

In our study, peripheral retinal screening at the end of vitrectomy in 106 patients who underwent MH surgery showed that 3 patients (2.8%) had retinal tear, 4 patients (3.8%) had retinal hole, and 10 patients (9.4%) had lattice degeneration. One of these patients had both lattice degeneration and retinal hole.

Previous studies, including those in postmortem eyes, have determined the prevalence of retinal tear to be 1-3.3%.^11,12,13,14^ Consistent with these studies, retinal tear was detected in 2.8% of patients who underwent MH surgery in our study.

Studies including postmortem eyes showed the prevalence of retinal hole was 2.4-4.4%.^15,16^ Similarly, in our study the prevalence of retinal hole was 3.8% among patients who underwent MH surgery.

The prevalence of the most common peripheral retinal degeneration, lattice degeneration, has been reported as 6-10.7%.^18,19,20^ In the present study, we also found the prevalence of lattice degeneration to be 9.4%.

In a study similar to ours that included 167 patients, the total prevalence of peripheral retinal tear + retinal hole + lattice degeneration accompanying MH was 33.8%, which was higher than the population average.^21^ In our study, the total prevalence of peripheral retinal tear + retinal hole + lattice degeneration was found to be 15.1%, which was similar to the population average.

In our study, the prevalence of retinal tear and hole in patients with MH, conditions believed to share the common etiology of VMT, was comparable to that in the general population, not higher. This will contribute to the literature, as it indicates that the vitreous does not have a uniform character, but may have different relationships with the macula and retina, and can develop different pathologies in these diseases.

As to why the detected retinal tears did not cause detachment, it may be due to the effect of other mechanisms that maintain adhesion between the retinal pigment epithelium and sensory retina, or there may not be sufficient vitreoretinal tractional force to cause detachment in every tear. Moreover, although the prevalence of retinal tear including in postmortem eyes was found to be 1-3.3%,^11,12,13,14^ considering that the prevalence of retinal detachment is 5-10.39 per 100,000,^25,26,27^ every tear may not lead to detachment.

### Study Limitations

Limitations of our study are the relatively small number of patients and the retrospective design.

## Conclusion

In summary, the prevalence of retinal tear and retinal hole in patients with macular hole, which are regarded as having similar etiologies, were similar to the population, suggesting that the pathologies involving the anterior and posterior vitreous may be different.

## Figures and Tables

**Table 1:**
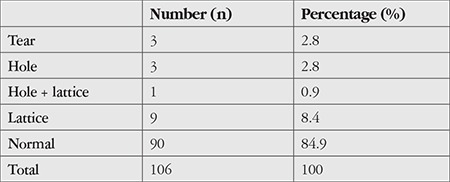
Peripheral retinal diseases associated with macular hole
